# Prognostic significance of post-preoperative tumor markers increments in patients with non-metastatic gastric cancer

**DOI:** 10.1007/s00432-023-05131-0

**Published:** 2023-07-10

**Authors:** Xiao-Dong Zhu, Li-Xiang Zhang, Pan-Quan Luo, Hai Zhu, Zhi-Jian Wei, A-Man Xu

**Affiliations:** 1 Department of General Surgery, The First Affiliated Hospital of Anhui Medical University, Jixi Road 218th, Shushan Street, Hefei, 230032 Anhui China; 2 Department of General Surgery, Anhui Provincial Hospital, Luyang Street, Hefei, 230036 Anhui China

**Keywords:** Gastric cancer, Carcinoembryonic antigen, Carbohydrate antigen 19-9, Prognosis, Nomogram

## Abstract

**Purpose:**

Carcinoembryonic antigen (CEA) and carbohydrate antigen 19-9 (CA19-9) are the most common tumor markers detected before and after gastric cancer (GC) surgery. However, the impact of post-preoperative CEA/CA19-9 increments on prognosis of GC remains unclear. In addition, there is no research incorporating post-preoperative CEA/CA19-9 increments into the prognostic model.

**Methods:**

Patients who underwent radical gastrectomy for GC at the First Affiliated Hospital of Anhui Medical University and Anhui Provincial Hospital from January 2013 to December 2017 were enrolled and divided into the discovery and validation cohort. Prognostic value of post-preoperative CEA/CA19-9 increments and preoperative CEA/CA199 levels were assessed by Kaplan–Meier log-rank analysis and compared by time-dependent receiver operating characteristic (*t*-ROC) curves. Multivariate Cox regression analysis was applied to establish the nomogram. The performance of the prognostic model was validated by the concordance index (C-index), calibration curve, and ROC curve analysis.

**Results:**

A total of 562 GC patients were included in this study. Overall survival (OS) rates decreased with an increasing number of incremental tumor markers after surgery. The *t*-ROC curves implied that the prognostic ability of the number of incremental post-preoperative tumor markers was superior to that of the number of positive preoperative tumor markers. Cox regression analysis suggested that the number of incremental post-preoperative tumor markers was an independent prognostic factor. The nomogram incorporated with the post-preoperative CEA/CA19-9 increments showed reliable accuracy.

**Conclusions:**

Incremental post-preoperative CEA/CA19-9 were indicator of poor prognosis of GC. The prognostic value of post-preoperative CEA/CA19-9 increments exceed that of preoperative CEA/CA19-9 levels.

## Introduction

Gastric cancer (GC) remains one of the most common malignant tumors worldwide. According to global cancer data statistics, there were over 1 million confirmed cases of GC resulting in more than 768,000 deaths in 2020, making GC the fifth most common malignancy and third leading cause of cancer related deaths in the word (Sung et al. 2021). Despite the continuous progress of surgical procedures and novel treatment, some patients still experience recurrence after radical surgery. Therefore, accurately predicting the prognosis is an important step in the management of each patient undergoing radical surgery. At present, the TNM staging system proposed by the American Joint Commission on Cancer (AJCC) is widely used in clinical practice to assess the prognosis of GC patients (Amin et al. 2017). However, due to the fact that this system does not consider prognostic factors other than tumor, lymph node, and metastasis, its accuracy has been questioned by scholars (Zhao et al. 2018; Röcken and Behrens 2015). Thus, in clinical practice, by combining other significant prognostic factors, such as serum tumor markers, the prognosis of GC patients can be better evaluated.

Carcinoembryonic antigen (CEA) and carbohydrate antigen 19-9 (CA19-9) have been confirmed as reliable tumor markers that can be used for the early diagnosis and postoperative monitoring of GC, and regular measurements are recommended (Wang et al. 2016b, a; Nam et al. 2013; Shimada et al. 2014). However, previous studies mainly focused on the prognostic value of preoperative and postoperative levels of CEA and CA19-9 (Lin et al. 2020; Suenaga et al. 2019). Currently, few studies have systematically analyzed the significances of post-preoperative CEA and CA19-9 increments for the prognosis of GC patients after radical gastrectomy. Therefore, the purpose of this study is to verify the significance of changes in serum tumor markers (CEA and CA19-9) before and after radical surgery for the prognosis of GC patients. Specially, we also determined whether preoperative tumor markers levels or post-preoperative tumor markers increments are more prognostic. Ultimately, we utilized the prognostic value of post-preoperative tumor markers increments to establish and validate a reliable nomogram to predict the outcome of GC patients.

## Materials and methods

### Study population

We retrospectively collected the clinical data of patients who underwent radical gastrectomy for GC at the First Affiliated Hospital of Anhui Medical University and Anhui Provincial Hospital from January 2013 to December 2017. The inclusion criteria included: (1) histologically confirmed primary gastric cancer; (2) no preoperative neoadjuvant treatment; (3) received R0 radical gastrectomy with systemic lymphadenectomy. Patients with distant metastasis, other malignant diseases and incomplete clinical information were excluded. Finally, a total of 562 patients were included in the study, with 408 patients from the First Affiliated Hospital of Anhui Medical University performed as the discovery cohort and 154 patients from Anhui Provincial Hospital performed as the validation cohort. This study complies with the ethical guidelines of the Helsinki Declaration of the World Medical Association and has been approved by the hospital ethics committee.

### Data collection

The clinical characteristics of patients were retrieved from medical record system, including gender, age, TNM stage, degree of differentiation, vascular invasion, pre- and postoperative serum tumor marker values, etc. Preoperative serum tumor markers were measured within one week before the surgery, while postoperative serum tumor markers value were the last measurement results within six months after gastrectomy but before the chemotherapy. Based on previous research (Sturgeon et al. 2010), the cutoff value of CEA and CA199 was 5 ng/ml and 37 U/ml, respectively. Besides, we calculated the prognostic nutritional index (PNI) and tumor-related inflammatory indicators, including neutrophil-to-lymphocyte ratio (NLR), lymphocyte to monocyte ratio (LMR), platelet to lymphocyte ratio (PLR) in accordance with previous literature (Tomás et al. 2022; Pikuła et al. 2022; Gao et al. 2018; Liu et al. 2017). Patients were divided into three groups respectively according to the number of positive tumor markers (CEA and CA199) before surgery and the number of incremental tumor markers after surgery. To further compare the predictive superiority between preoperative tumor markers levels and post-preoperative tumor markers increments, we divided patients into four groups based on whether preoperative tumor markers were positive and whether postoperative tumor markers were elevated.

### Follow-up investigation

All patients underwent postoperative follow-up according to the guidelines. Survival status were obtained by telephone, text message, and other means. The follow-up endpoint was set to May 2020. Overall survival (OS) was defined as the time from surgery to death of any cause.

### Statistical analysis

All data were analyzed by IBM SPSS Statistics 26.0 software, GraphPad Prism 9 and R software, version 4.2.1. Survival curves were assessed by the Kaplan–Meier method and compared by the log-rank test among different groups. We used the time-dependent receiver operating characteristic (*t*-ROC) curves to compare the prognostic ability of the preoperative tumor markers levels and post-preoperative tumor markers increments in discovery and validation cohort. The cutoff value of the continuous variables was obtained by using the package of *survminer* in R software, version 4.2.1. Other prognostic factors were screened using the univariate and multivariate Cox proportional hazard regression in the discovery cohort, to establish the prognostic model. Based on the results of multivariate Cox proportional hazard regression, a nomogram was formulated. The performance of the prognostic model was verified through the concordance index (C-index), calibration, and ROC curve analysis on 1,000 bootstrap samples. In addition, we validated the reliability of nomogram in validation cohort. Two-tailed P value < 0.05 was considered statistically significant in all tests.

## Results

### Patient characteristics

In total, 562 GC patients were included in this study. Among them, 408 patients from the First Affiliated Hospital of Anhui Medical University served as the discovery cohort and 154 patients from Anhui Provincial Hospital served as the validation cohort.

The discovery cohort included 275 (67.4%) males and 133 (32.6%) females, 230 (56.4%) of whom were over 60 years old. According to the TNM staging system, there were 46 (11.3%), 107 (26.2%), and 255 (62.5%) of patients diagnosed as stage I, II, and III GC, respectively. Specifically, 34 (8.3%) patients were in T1 stage, 44 (10.8%) patients were in T2 stage, 75 (18.4%) patients were in T3 stage, and 255 (62.5%) patients were in T4 stage. A total of 309 patients had lymph node metastasis, including 78 (19.1%) patients with N1 stage, 123 (30.1%) patients with N2 stage, 108 (26.5%) patients with N3 stage. Additionally, 204 (50.0%) cases showed poor or undifferentiated differentiation, while another 192 (47.1%) cases showed moderate differentiation, with only 12 (2.9%) cases showing well differentiated. Vascular invasion was observed in 101 (24.8%) cases. According to data, there were 288 (70.6%) patients with no positive tumor markers, 101 (24.7%) patients with one and just 19 (4.7%) with two positive tumor markers before the surgery. Meanwhile, there were 181 (44.4%) patients with no incremental tumor markers, 134 (32.8%) patients with one and 93 (22.8%) with two incremental tumor markers after surgery. At the end of the follow-up, 172 (42.2%) patients died (Table 1).

**Table 1:** Demographics and clinicopathologic features of patients

Variables	Patients (*N* = 408)
*Gender*	
Male	275 (67.4%)
Female	133 (32.6%)
*Age*	
< 60	178 (43.6%)
≥ 60	230 (56.4%)
*Clinical stage* (*pTNM*)	
I	46 (11.3%)
II	107 (26.2%)
III	255 (62.5%)
*T stage*	
T1	34 (8.3%)
T2	44 (10.8%)
T3	75 (18.4%)
T4	255 (62.5%)
*N stage*	
N0	99 (24.3%)
N1	78 (19.1%)
N2	123 (30.1%)
N3	108 (26.5%)
*Differentiation*	
Well	12 (2.9%)
Moderate	192 (47.1%)
Poor and undifferentiated	204 (50.0%)
*Vascular invasion*	
Positive	101 (24.8%)
Negative	307 (75.2%)
*Number of positive tumor markers before surgery*	
0	288 (70.6%)
1	101 (24.7%)
2	19 (4.7%)
*Number of incremental tumor markers after surgery*	
0	181 (44.4%)
1	134 (32.8%)
2	93 (22.8%)
*Survival status*	
Alive	236 (57.8%)
Dead	172 (42.2%)

### Prognostic significance of preoperative tumor markers levels and post-preoperative tumor markers increments

**Fig. 1:**
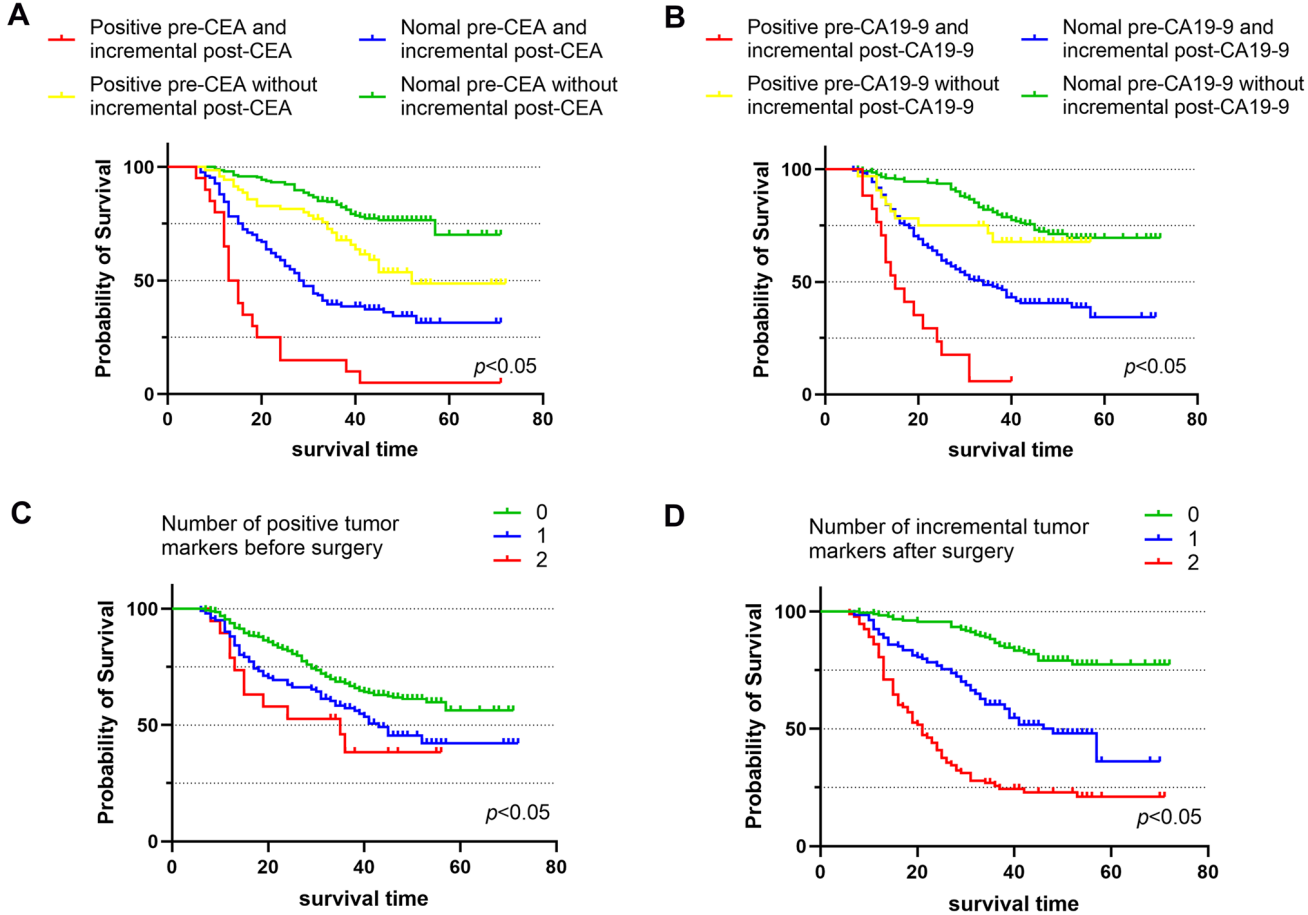
Kaplan–Meier analysis for patients with gastric cancer (GC) in discovery cohort. **A** and **B** are stratified by preoperative tumor markers (pre-CEA and pre-CA19-9) levels and postoperative tumor markers (post-CEA and post-CA19-9) increments. **C** and **D** are stratified by the number of positive tumor markers before surgery and the number of incremental tumor markers after surgery. CEA, carcinoembryonic antigen; CA19-9, carbohydrate antigen 19–9

**Fig. 2:**
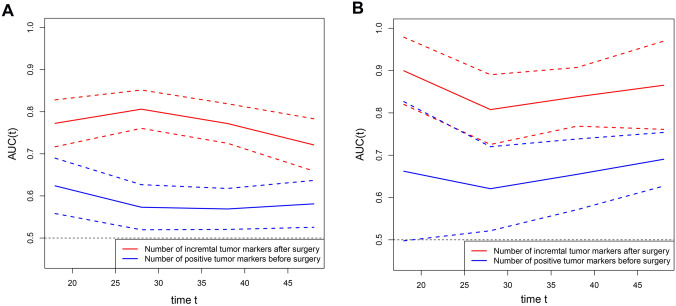
Time-dependent ROC curves for tumor makers. Time-dependent ROC curves for number of positive tumor markers before surgery and the number of incremental tumor markers after surgery in discovery cohort **(A)** and validation cohort **(B)**. The horizontal axis represents the month after surgery, and vertical axis represents the estimated area under the curve (AUC) for survival at the corresponding time. Red and blue solid lines represent the estimated AUCs of the number of incremental tumor markers after surgery and the number of positive tumor markers before surgery, respectively, and broken lines represent the 95% confidence intervals of each AUC. ROC, receiver operating characteristic

The Kaplan–Meier survival curves were used to evaluate the effects of positive preoperative tumor markers and post-preoperative tumor markers increments on survival time of GC. As mentioned in the experimental method, we divided patients into four groups, respectively, based on whether CEA or CA199 was positive before surgery and incremental after surgery. The Kaplan–Meier survival curves showed that patients with normal preoperative tumor markers and without postoperative increment had the best prognosis, while patients with positive preoperative tumor markers and postoperative increment had worst prognosis (*p* < 0.001) (Fig. 1A, B). Our results showed that the OS rate decreased with an increasing number of positive tumor markers before surgery and the number of incremental tumor markers after surgery (Fig. 1C, D). Similarly, in univariate Cox regression analysis, there was a significant correlation between the number of positive preoperative tumor markers and the number of incremental postoperative tumor markers with OS (Table 2). The *t*-ROC curves implied that the prognostic ability of the number of incremental post-preoperative tumor markers was superior to that the number of positive preoperative tumor markers both in discovery and validation cohort (Fig. 2).

**Table 2:** Univariate and multivariate analysis for overall survival (OS) of gastric cancer (GC) patients in discovery cohort

Variables	Univariate analysis		Multivariable analysis	
	OR (95%CI)	*p*-Value	OR (95%CI)	*p*-Value
*Gender*				
Male	Reference	–		
Female	1.223 (0.896–1.670)	0.205		
*Age*				
< 60	Reference	–		
≥ 60	1.249 (0.920–1.697)	0.154		
*T stage*				
T1, T2	Reference	–	Reference	–
T3	3.234 (1.565–6.683)	**0.002**	2.571 (1.201–5.505)	**0.015**
T4	5.609 (2.949–10.669)	< **0.001**	3.887 (1.972–7.662)	< **0.001**
*N stage*				
N0, N1	Reference	–	Reference	–
N2	2.348 (1.571–3.509)	< **0.001**	1.599 (1.052–2.429)	**0.028**
N3	4.840 (3.298–7.103)	< **0.001**	1.763 (1.133–2.742)	**0.012**
*Differentiation*				
Moderate, well	Reference	–	Reference	–
Poor, undifferentiated	2.087 (1.530–2.848)	< **0.001**	1.509 (1.091–2.089)	**0.013**
*Vascular invasion*				
Negative	Reference	–	Reference	–
Positive	2.093 (1.529–2.866)	< **0.001**	1.107 (0.791–1.550)	0.554
*Number of incremental tumor markers after surgery*				
0	Reference	–	Reference	–
1	3.199 (2.119–4.828)	< **0.001**	2.502 (1.633–3.833)	< **0.001**
2	7.818 (5.194–11.768)	< **0.001**	5.747 (3.625–9.114)	< **0.001**
*Number of positive tumor markers before surgery*				
0	Reference	–		
1	1.573 (1.130–2.189)	**0.007**		
2	2.147 (1.154–3.995)	**0.016**		
*PNI*				
> 46.8	Reference	–	Reference	–
≤ 46.8	1.968 (1.456–2.658)	< **0.001**	1.316 (0.940–1.842)	0.109
*NLR*				
≤ 2.08	Reference	–	Reference	–
> 2.08	2.084 (1.523–2.851)	< **0.001**	1.742 (1.218–2.492)	**0.002**
*PLR*				
≤ 140.09	Reference	–	Reference	–
> 140.09	1.863 (1.377–2.521)	< **0.001**	1.098 (0.772–1.561)	0.604
*LMR*				
> 4.24	Reference	–	Reference	–
≤ 4.24	1.673 (1.241–2.257)	**0.001**	0.911 (0.645–1.286)	0.595

### Univariate and multivariate analysis of factors related to OS in discovery cohort

To identify the independent prognostic factors for OS in discovery cohort, we performed univariate and multivariate Cox regression analyses. In univariate analyses, T stage, N stage, degree of tumor differentiation, vascular invasion, the number of incremental post-preoperative tumor markers, the number of positive preoperative tumor markers, PNI, NLR, PLR, and LMR were associated with OS. Subsequently, considering the *t*-ROC curves result, multivariate analysis results confirmed that T stage [T3, HR: 2.571 (1.201–5.505), *p* = 0.015; T4, HR: 3.887 (1.972–7.662), *p* < 0.001], N stage [N2, HR: 1.599 (1.052–2.429), *p* = 0.028; N3, HR: 1.763 (1.133–2.742), *p* = 0.012], degree of tumor differentiation [HR: 1.509 (95%CI1.091–2.089), *p* = 0.013], the number of incremental post-preoperative tumor markers [*n* = 1, HR: 2.502 (1.633–3.833), *p* < 0.001; *n* = 2, HR: 5.747 (3.625–9.114), *p* < 0.001], and NLR [> 2.08, HR: 1.742 (1.218–2.492), *p* = 0.002] were significant independent prognostic factors for OS (Table 2).

### Development and verification of prognostic nomogram

**Fig. 3:**
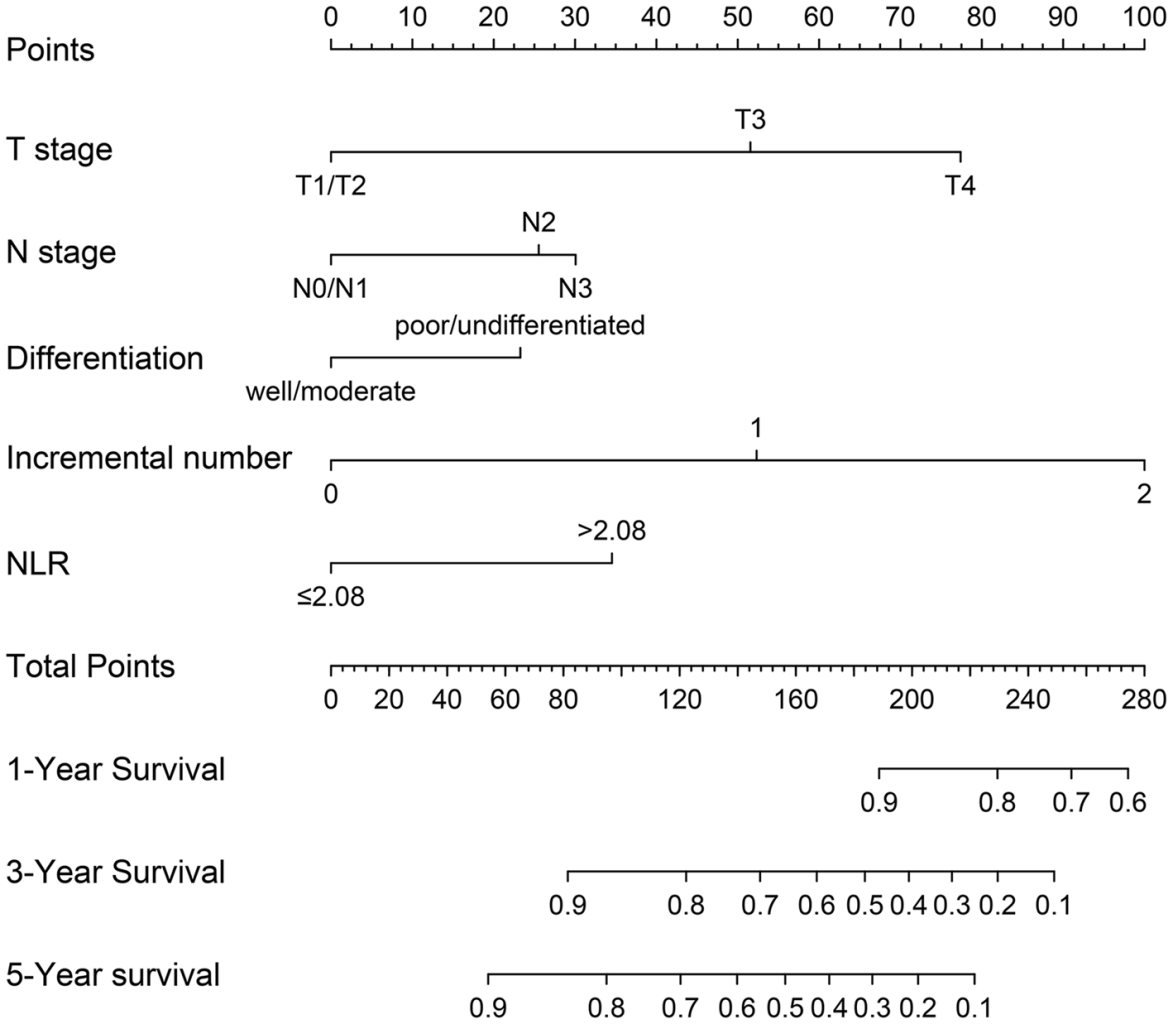
Prognostic nomogram for prediction of the 1-year, 3-year, and 5-year overall survival (OS) of patients with gastric cancer (GC). NLR, neutrophil-to-lymphocyte ratio; incremental number, the number of incremental tumor markers after surgery

**Fig. 4:**
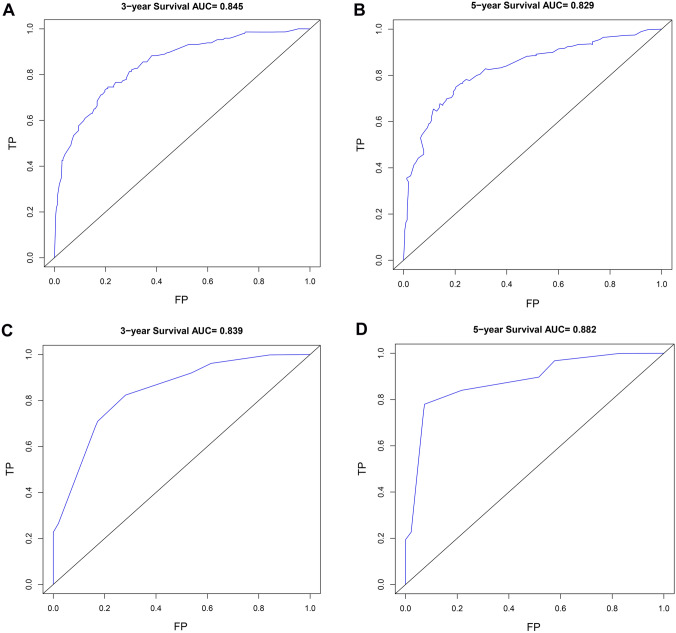
ROC curves of the predictive nomogram for prediction of the overall survival (OS) of patients with gastric cancer (GC). **A**, **B** represent ROC curves of the model for predicting patient survival at 3 years and 5 years in discovery cohort. (C, D) represent ROC curve of the model for predicting patient survival at 3-year and 5-year in validation cohort. *ROC* receiver operating characteristic, *AUC* area under the curve. *TP* true positive, *FP* false positive

**Fig. 5:**
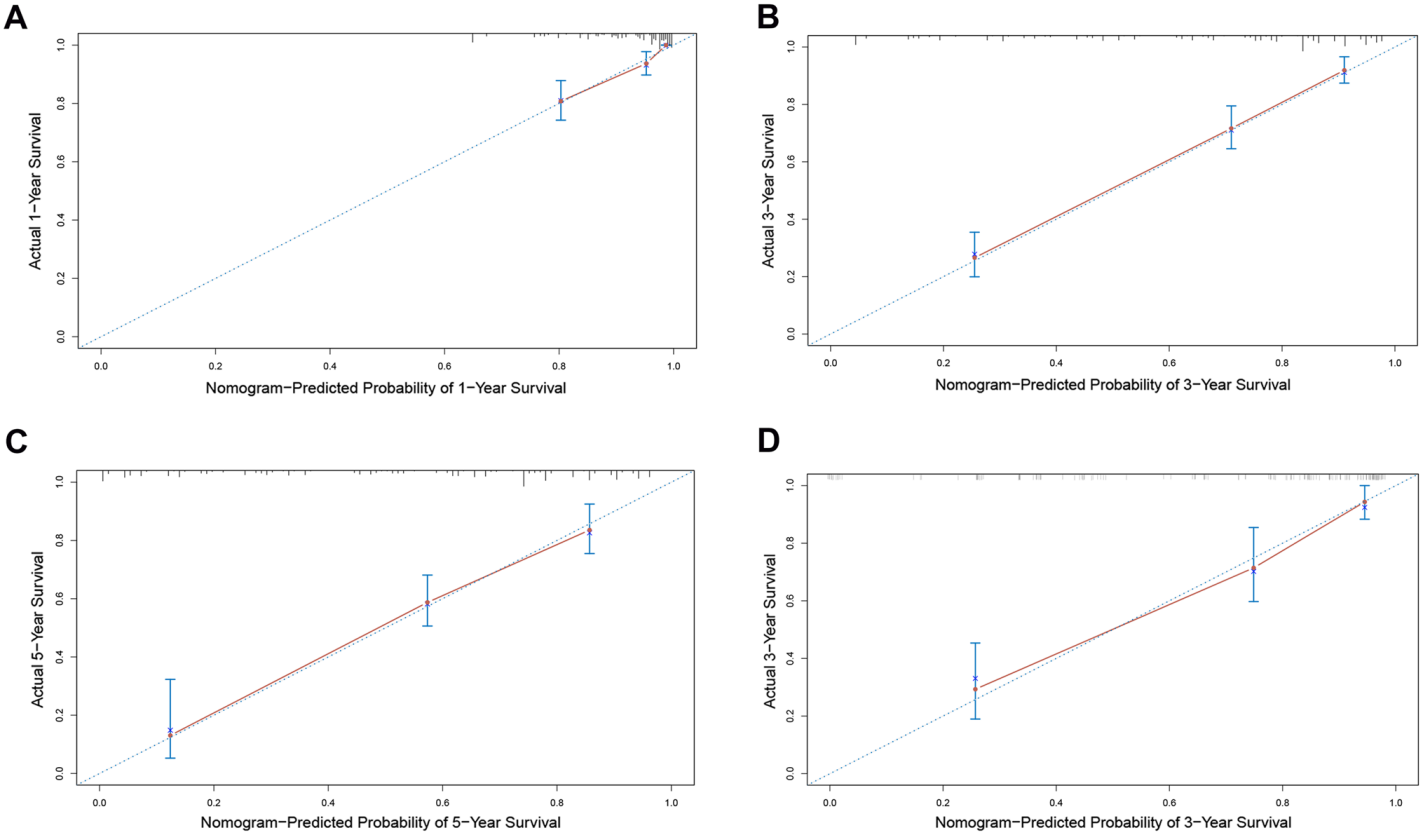
Calibration curves for nomogram predictions. **A–C** represent calibration curves of the model predicting 1-year, 3-year, and 5-year overall survival (OS) in discovery cohort, respectively. **D** represents calibration curves of the model predicting 3-year OS in validation cohort

Based on the results of cox regression analysis, the prognostic nomogram integrating all independent factors was established (Fig. 3). The OS rate of an individual patients was calculated based on the nomogram. Assign a corresponding risk score to each prognostic feature based on the actual value of the variable, and sum up the risk scores of all variables to obtain the 1-, 3-, 5-year OS predictions based on the bottom scale. The predictive performance of the nomogram was evaluated though the C-index. The C-index for the nomogram was 0.794 (95% CI, 0.765–0.823). Additional, ROC plot (Fig. 4A, B) showed the accuracy of the model in predicting the 3-, 5-year OS. The calibration plot for the 1-, 3-, 5-year OS rate showed favorable agreement between the predicted results of the nomogram and actual observation (Fig. 5A–C). In external validation, the C-index of nomogram for predicting the OS rate of the validation cohort was 0.821 (95%CI, 0.766 to 0.876). Besides, calibration curve for predicting 3-year survival confirmed a high degree of fit between prediction and observation in validation cohort. (Fig. 5D). According to the ROC plot (Fig. 4C, D), the nomogram also showed satisfying predictive accuracy in validation cohort.

## Discussion

CEA and CA19-9 are widely performed as indicators for early diagnosis and postoperative follow-up of GC in clinical practice. Studies have shown that a single tumor marker has lower sensitivity and specificity in the diagnosis and prognosis of cancer compared to the combination of multiple tumor markers (Wang et al. 2016a,b; He et al. 2013; Toyoda et al. 2012). Besides, the detection of serum CEA and CA19-9 have a high priority in terms of cost, invasiveness, and availability; therefore, we examined the prognostic value of the combination of CEA and CA19-9 for GC through multicenter data. Most previous studies focused on the level of pre- and postoperative tumor markers, and there is still no consensus on which can better predict the prognosis of GC (Kodera et al. 1996; Jing et al. 2020; Zhang et al. 2017). However, there are few researches shown the impact of the increment of postoperative CEA and CA19-9 on the prognosis compared to the preoperative levels. In our study, we assessed the prognostic value of the increment of postoperative CEA and CA19-9 in non-metastatic GC and confirmed its superiority of prognosis compare to the level of preoperative CEA and CA19-9. In addition, we utilized independent prognostic factors including T stage, N stage, tumor differentiation, NLR, and number of incremental tumor markers after surgery to establish the predictive nomogram. The nomogram showed satisfied prediction accuracy according to the internal and external validation.

Currently, several researches have shown that pre- and postoperative tumor markers are associated with prognosis of GC, but there had always been a contradiction which had better predictive reliability compared to the another. Lin et al. (2020) and Uda et al. (2018) found that preoperative tumor markers had better prognostic value than postoperative tumor markers. Nevertheless, Suenaga et al. (2019) pointed out that the postoperative CEA and CA19-9 were independent prognostic factors for patients with stage II/III GC, while the preoperative values were not. Such opposing conclusion might be related to their different inclusion criteria and variable classification methods. However, there were only 46 (11.2%) and 29 (7.1%) patients, respectively, with positive CEA and CA19-9 in the discovery cohort. Limited by the number of patients with positive postoperative tumor markers, we could not compare the prognostic value of postoperative tumor markers levels with post-preoperative tumor markers increments.

The increment of postoperative tumor markers might be related to the residual minute cancer tissue during surgery or the presence of micrometastasis that cannot be recognized by imaging examination (Toyoda et al. 2012; Kanda et al. 2018). In our group study, we found that prognosis of patients with positive preoperative CEA and without postoperative increment was significantly better than that of patients with negative preoperative CEA and postoperative increment, but was inferior to those with negative CEA and without postoperative increment (Fig. 1A). Meanwhile, the analysis of CA19-9 had similar results, except that there was no significant difference in the survival curve between patients with positive preoperative CA19-9 and without postoperative increment and those with negative preoperative CA19-9 and without postoperative increment (Fig. 1B). According to these conclusions, regardless of the level of preoperative tumor makers, the increment of postoperative tumor markers implied a worse prognosis. Based on the *t*-ROC curves, we confirmed that post-preoperative tumor markers increment had a better prognostic ability for GC. In the end, we validated the feasibility of the addition of postoperative tumor markers increment into the prognostic models. Lin et al. (2018) suggested that the including of CEA/CA19-9 level in AJCC TNM staging system could improve the prediction accuracy of stage III GC outcome. Back in 2000, the Working Group of AJCC recommended incorporating serum CEA level into the TNM staging of colon cancer (Compton et al. 2000). The effectiveness of this improvement method has been verified by Zhou et al. (2021). This was the first study to incorporate the increment of postoperative CEA/CA19-9 into prognostic model for predicting OS in patients with GC. Inflammation ratios were regarded as basilic feature of cancer. NLR had been reported as a prognostic factor and one of the reference indicators for postoperative adjuvant therapy in GC (Li et al. 2017; Nechita et al. 2022; Miyamoto et al. 2018). Analogously, we validated that NLR is an independent predictor for GC outcome.

Current guidelines recommended a regular detection of CEA/CA19-9 every 3–6 months in patients after radical resection of GC (Lordick et al. 2022; Wang et al. 2021). However, guidelines do not provide an indication for individual follow-up and adjuvant therapy intensity. Our results indicated that the increment of postoperative CEA/CA19-9 may inform the frequency and degree of follow-up. For instance, once the increment of CEA/CA19-9 was detected after surgery, more detailed examinations as CT should be considered to identify recurrence, and more frequent testing of serum CEA/CA19-9 is recommended.

There are several limitations of this study. First, the inherent limitation and biases of retrospective research. For example, part of patients did not accept the blood tests after surgery. Patients who accepted postoperative tumor markers testing were more inclined toward advanced GC. Second, due to the relatively short follow-up duration, the credibility of the nomogram in predicting 5-year OS might be affected. Besides, the time limit for measuring postoperative tumor markers was not controlled. The span of within six months after surgery and before starting chemotherapy was a bit broad. Finally, we did not evaluate the prognostic value of postoperative tumor markers levels compared to post-preoperative tumor markers increments.

In conclusion, the prognostic value of post-preoperative CEA/CA19-9 increments exceeds that of preoperative CEA/CA19-9 levels. The prognostic nomogram based on post-preoperative CEA/CA19-9 increments and other prognostic factors could provide effective information for postoperative management of GC patients to improve their prognosis. The detection of postoperative tumor markers requires more attention. Patients with incremental CEA/CA19-9 tend to worse outcome, and more aggressive surveillance strategy and treatments should be implemented.

## Data Availability

The data used and/or analyzed during the current study were obtained from the Department of Gastrointestinal Surgery, the First Hospital of Anhui University. The data are available from the corresponding author on reasonable request.
